# Effectiveness of recombinant *Escherichia coli* on the production of (*R*)-(+)-perillyl alcohol

**DOI:** 10.1186/s12896-020-00662-7

**Published:** 2021-01-08

**Authors:** Chao Sun, Xianjuan Dong, Rubing Zhang, Congxia Xie

**Affiliations:** 1 A State Key Laboratory Base of Eco-Chemical Engineering, College of Chemistry and Molecular Engineering, Qingdao University of Science and Technology, 53 Zhengzhou Rd., Qingdao, 266042 China; 2 CAS Key Laboratory of Bio-based Materials, Qingdao Institute of Bioenergy and Bioprocess Technology, Chinese Academy of Sciences, 189 Songling Rd., Qingdao, 266101 China

**Keywords:** Limonene, Perillyl alcohol, *Escherichia coli*, Mevalonate pathway, Microbial production

## Abstract

**Background:**

(*R*)-(+)-perillyl alcohol is a naturally oxygenated monoterpene widely used as the natural flavor additives, insecticides, jet fuels and anti-cancer therapies. It was also readily available monoterpene precursors. However, this natural product is present at low concentrations from plant sources which are not economically viable. Therefore, alternative microbial production methods are rapidly emerging as an attractive alternative to make (*R*)-(+)-perillyl alcohol production more sustainable and environmentally friendly.

**Results:**

We engineered *Escherichia coli* to possess a heterologous mevalonate (MVA) pathway, including limonene synthase*,* P-cymene monoxygenase hydroxylase and P-cymene monoxygenase reductase for the production of (*R*)-(+)-perillyl alcohol. The concentration of (*R*)-(+)-limonene (the monoterpene precursor to (*R*)-(+)-perillyl alcohol) reached 45 mg/L from glucose. Enhanced (*R*)-(+)-perillyl alcohol production was therefore achieved. The strain produced (*R*)-(+)-perillyl alcohol at a titer of 87 mg/L and a yield of 1.5 mg/g glucose in a 5 L bioreactor fed batch system.

**Conclusions:**

These datas highlight the efficient production of (*R*)-(+)-perillyl alcohol through the mevalonate pathway from glucose. This method serves as a platform for the future production of other monoterpenes.

## Background

Perillyl alcohol is a natural monoterpene that exists in two optical forms. (*R*)-(+)-perillyl alcohol is produced in perilla leaves, citrus, lemon and lavender [1–3] and has extensive applications. It serves as a natural flavor additive for food, as an insecticide in agricultural fields, as a jet fuel in aviation fields, and as a healing agent for anti-cancer therapeutics [4–7]. The bioconversion of readily available monoterpene precursors (such as (*R*)-(+)-limonene) are recognized as valuable oxygenated derivatives [8]. Up to 70–97% of (*R*)-(+)-limonene is present in citrus oils [9], produced to levels that exceed 60,000 t per year [10]. Many terpenes are synthesized from limonene, such as perillyl alcohol, carvone and α-terpineol. The enantiomers of carvone cost US $ 30–60 per kg; whilst (*S*)-(−)- and (*R*)-(+)-perillyl alcohol cost US $4500/kg [7, 11]. As the chemical synthesis of perillyl alcohol is of high cost and leads to environmental pollution, the biosynthesis of perillyl alcohol from renewable carbon sources is regarded as an economically feasible industrial process. New and more effective synthesis procedures are however required.

(*R*)-(+)-perillyl alcohol can be synthesized in *Escherichia coli* [12], *Pseudomonas putida* [5, 13–16], *Mortierella minutissima* [17], *Fusarium verticilloides* [18], *Aspergillus* strain [19, 20] from the biotransformation of (*R*)-(+)-limonene to (*R*)-(+)-perillyl alcohol (Table. 1). The recombinant *Pseudomonas taiwanensis* VLB120 harboring *cymAa* and *cymAb* from *Pseudomonas putida* can convert (*R*)-(+)-limonene to (*R*)-(+)- perillic alcohol [15] (Table 1). The maximum yield of (*R*)-(+)-perillyl alcohol was 258.1 mg/L for 3 days using *Mortierella minutissima* cultivation containing 0.5% (*R*)-(+)-limonene at 15 °C [17]. This produced the highest levels of (*R*)-(+)-perillyl alcohol currently reported. It should be as a substrate for the above microbial transformation reaction, but the raw materials were not easily obtained [22]. Metabolic engineering for natural compound production can be enhanced through gene modifications that increase enzyme activity. Many terpenoids have been produced at high titers by metabolic engineering, including monoterpenes, sesquiterpenes, diterpenes and tetraterpenes [23–29].

**Table 1:** Biosynthesis of (*R*)-(+)-perillyl alcohol l by microbial transformation

Host	Stereo isomer	Product	Titers	Yield	Carbon source and cultivations	Recovery method	Reference
*Escherichia coli*	(+)	perillyl alcohol	0.51 mg/L	NA	Glucose, (*R*)-(+)-limonene, sh-ake flask (24 h)	Closed culture flask	[12, 21]
*Pseudomonas taiwanensis* VLB120DC	(+)	perillyl alcohol	NA	100umol/g_cdw_	(*R*)-(+)-limonene, shake flask (1 h)	Closed culture flask	[15]
*Aspergillus cellulosae* M-77	(+)	perillyl alcohol	NA	NA	(*R*)-(+)-limonene, shake flask	Closed culture flask	[20]
*Mortierella minutissima* 01	(+)	perillyl alcohol	258.1 mg/L	NA	(*R*)-(+)-limonene, shake flask (72 h)	Closed culture flask	[17]

**Fig. 1:**
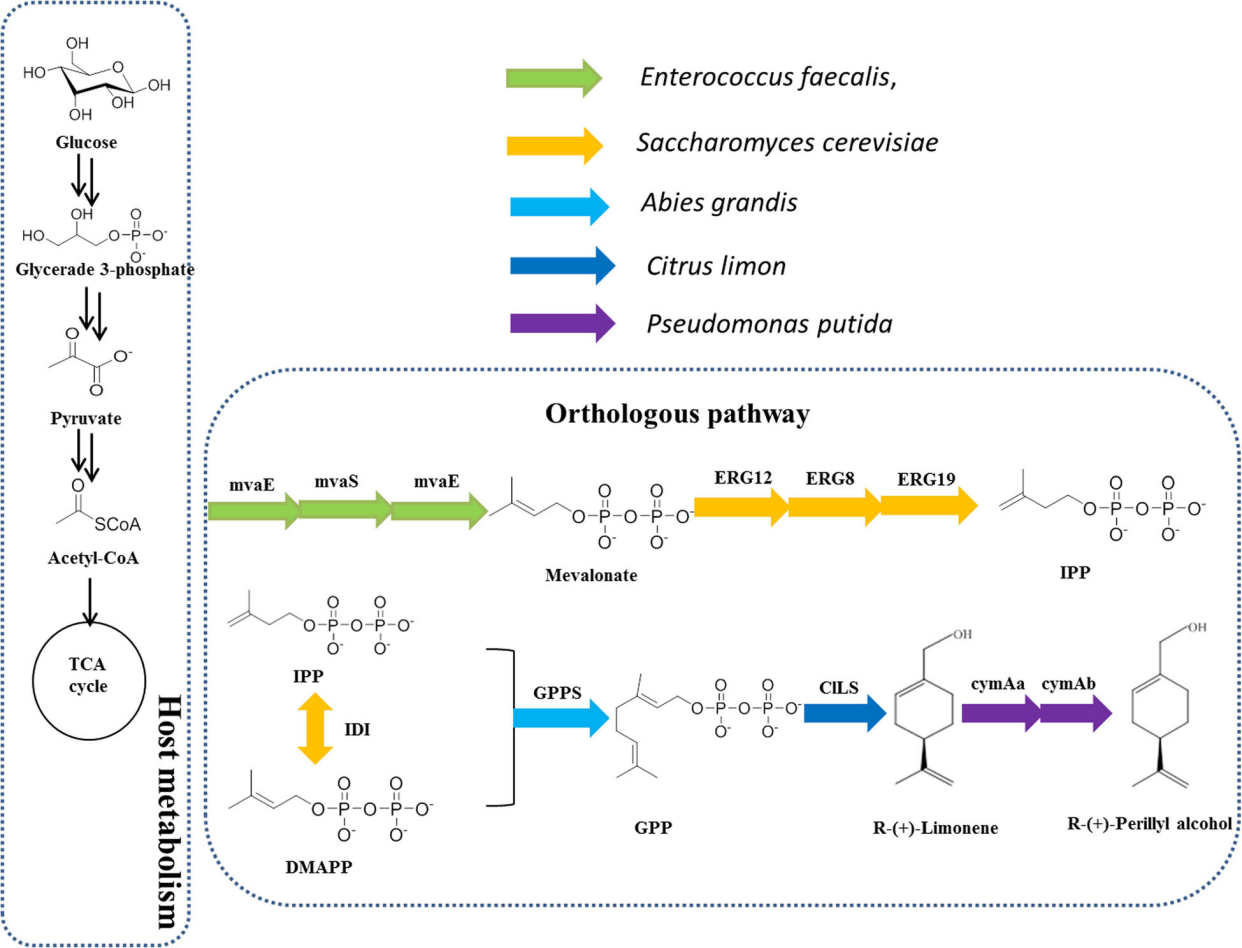
(*R*)-(+)-perillyl alcohol production via the MVA pathway. Enzymes are depicted in the legend

Isopentenyldiphosphate (IPP) and its isomer dimethylallyl diphosphate (DMAPP) form the building blocks of monoterpenes, sesquiterpenes and diterpenes during synthesis, the precursors of which include geranyl pyrophosphate (GPP), farnesylpyrophosphate (FPP), and geranylgeranyl pyrophosphate (GGPP), respectively. These precursors are derived from mevalonate (MVA) and are catalyzed by geranyl pyrophosphate diphosphate synthase (GPPS) (Fig. 1). However, the microbial mediated production of monoterpenes is limited by toxicity [29] and poor GPPS expression [30, 31]. In this study, we initially engineered *E. coli* to produce (*R*)-(+)-perillyl alcohol from glucose by the heterologous expression of P-cymene monoxygenase hydroxylase (CymAa) and a P-cymene monoxygenase reductase (CymAb) (Fig. 1). Genetic modifications to enhance the production of (*R*)-(+)-perillyl alcohol were also performed. These included the codon optimization of *cymAa*, *cymAb* and *ClLS* and exogenous *GPPS* expression. The highest performing strain SC04 was then cultured under fed-batch conditions for the assessment of its potential for large-scale production.

## Results

### Perillyl alcohol production from limonene

**Fig. 2:**
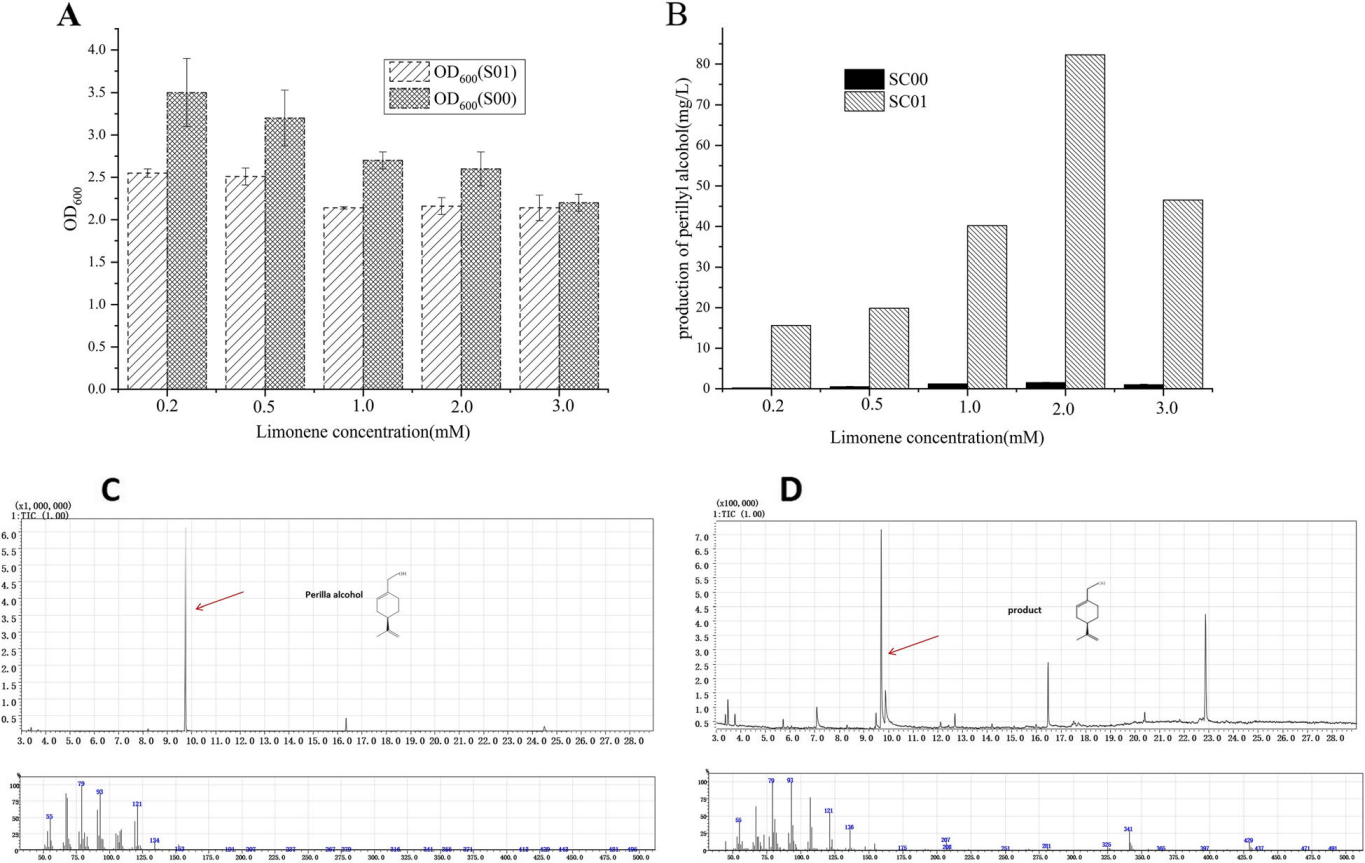
Effects of limonene concentration on the activity of SC00 and SC01. When the OD_600_ reached 0.6, samples were induced at 30 °C for 24 h using 0.2 mM IPTG and different contents of (*R*)-(+)-limonene (0.1 mM, 0.2 mM, 0.5 mM, 1.0 mM, 2.0 mM). **a** OD_600_ and (**b**) titres (mg/L) over different concentration of limonenes for 2 strains. **c** standard substance perillyl alcohol. **d** the fermentation product with 2 mM (*R*)-(+)-limonene for SC01. Error bars: SD from three independent cultivations

It is well established that during (*S*)-(−)-perillyl alcohol production, titers can be increased by 100 mg/L using *Escherichia coli* [32]. Many terpenoids have been produced to high titers through metabolic engineering, including monoterpenes, sesquiterpenes, diterpenes and tetraterpenes. Limonene hydroxylase from *Bacillus stearothermophilus* BR 388 (LHBS), was proved to produce (*R*)-(+)- perillyl alcohol 0.51 mg/L with the substrate (*R*)-(+)-limonene [12, 21]. *LHBS* was ligated into pET28a(+) to create pSC00, meanwhile, *cymAa* and *cymAb* were created pSC01 in Table S1. Limonene is toxic to most microorganisms and its concentrations regulate the growth of BL21 (DE3). It was therefore important to assess the optimal concentrations of limonene for fed-batch production. Different (*R*)-(+)-limonene concentrations were assessed (0.2 mM to 3.0 mM) in growth assays. With the increasing of (*R*)-(+)-limonene concentration, cell growth was inhibited. At (*R*)-(+)-limonene concentrations of 1.0 mM, the OD_600_ decreased from 2.5 to 2.0 for SC01 (Fig. 2a). When (*R*)-(+)-limonene was used at 2 mM, the maximum production was 86.9 mg/L and the conversion yield of perillyl alcohol was 30% for SC01 (Fig. 2b). For another strain SC00, it produced the low concentration of 1.5 mg/L, and cell growth was suppressed (Fig. 2b). In the later study, the strain SC01 was used as the research basis.

Products were identified by GC-MS after organic extraction by ethyl acetate. As shown in Fig. 2c and d, control assays with extracts of *E. coli* lacking a cDNA clone did not yield (*R*)-(+)-perillyl alcohol, whilst *E. coli* carrying *cymAa* and *cymAb* from *Pseudomonas putida* produced (*R*)-(+)-perillyl alcohol to detectable quantities. The biosynthetic pathway for (*R*)-(+)-perillyl alcohol production was successfully constructed using (*R*)-(+)-limonene as a substrate.

#### Limonene production from glucose

We optimized the yields of limonene to improve the production of perillyl alcohol. pTrcHis2B and pET-28a (+) were assessed. pYJM14 contains *ERG8, ERG19, IDI* from *Saccharomyces cerevisiae* [33]. pSC02 contains geranyl pyrophosphate synthase *GPPS* from *Abies grandis* that converts IPP/DMAPP to GPP, and (*R*)-(+)-limonene synthase *CILS* from *Citrus limon* that converts GPP to limonene [34] (Table S1). SC02 was cultured in fermentation medium under sealed shake-flask conditions.

**Fig. 3:**
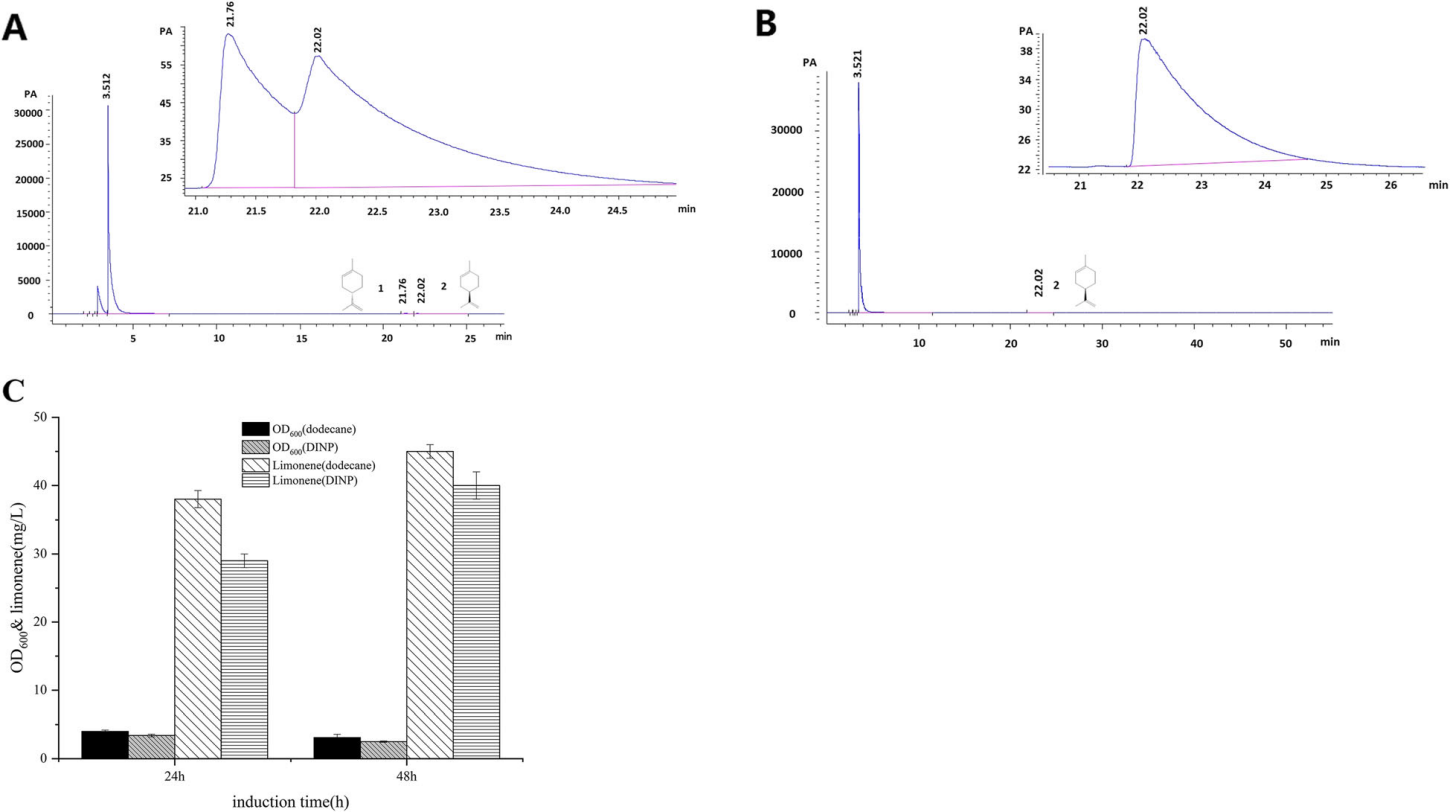
Limonene production using the SC02 strain. **a** The enantiomeric distribution of limonene was analyzed using the Agilent Technologies 7890B GC System on a Cyclodex B column (30 m × 0.25 mm internal diameter; film thickness = 0.25 μm). GC conditions were as follows: 50 °C hold, 2 °C/min ramping to 160 °C; carrier: high-purity helium, linear velocity: 1 ml/min; temperature of the injector: 250 °C; split ratio: 1:20. Compounds of interest: (*S*)-(−)-limonene at 21.2 min and (*R*)-(+)-limonene at 22.02 min. **b** The fermentation broth was mixed and centrifuged, and the organic layer was analyzed. **c** The total production of Limonene was measured after 24 h and 48 h of IPTG induction and 10% n-dodecane or DINP induction

The levels of (*R*)-(+) limonene in the culture media were plotted against known concentrations of (*R*)-(+)-limonene. The (*R*)-(+)-limonene concentration of SC02 reached 38 mg/L and 45 mg/L (mg limonene per liter of culture, the same as follows) with 0.2 mM IPTG and 10% n-dodecane overlay at 24 h and 48 h of induction. When 10% DINP (diisononyl phthalate) was used instead of the n-dodecane overlay, the limonene titers were 29 mg/L and 40 mg/L respectively at 24 h and 48 h (Fig. 3c). The enantiomer forms produced by the strain cultures were analyzed using GC with a Cyclosil-B column, and the production of (*R*)-(+)-limonene by SC02 was confirmed (Fig. 3a and b).

### Microbial perillyl alcohol production using the MVA pathway

**Fig. 4:**
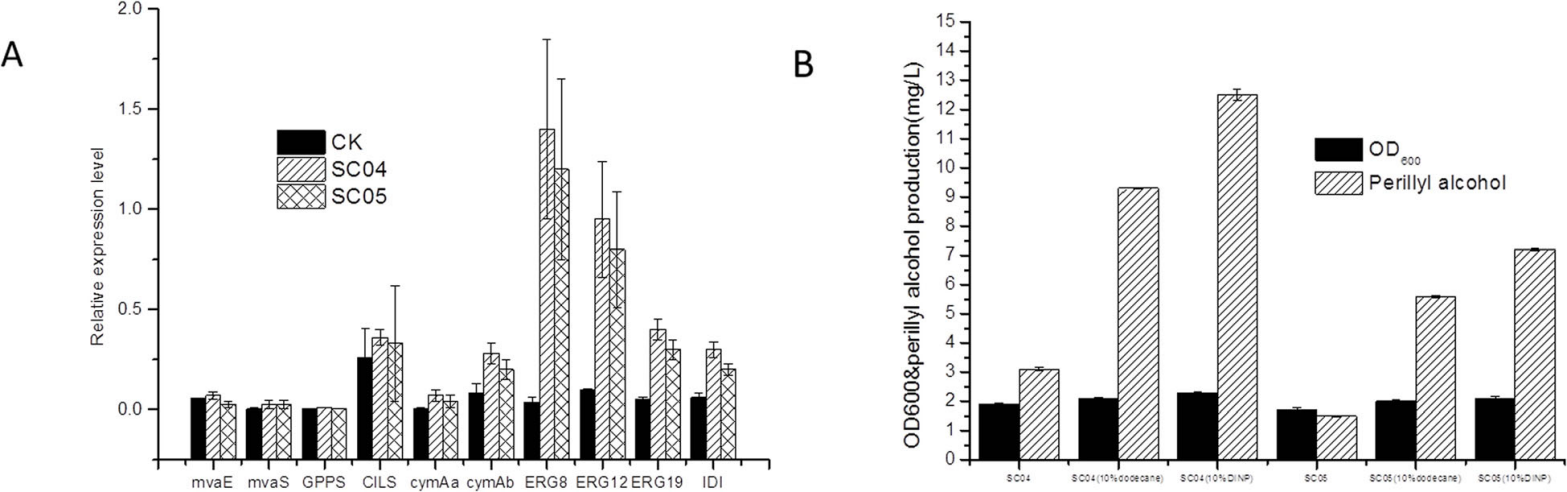
(*R*)-(+)-Perillyl alcohol production using SC04 and SC05 strains. **a** relative expression levels in SC04 and SC05 engineered for (*R*)-(+)-perillyl alcohol production. Data was for 24 h in LB medium with 0.2 mM IPTG and 20 g/L glucose. Gene expression was calculated using the 2^-△△CT^. system and are shown as the mean ± SD (*n* ≥ 3). Identical letters indicate no significant differences (*p* < 0.05). (5) Total production of (*R*)-(+)-perillyl alcohol was measured at 48 h after induction with 0.2 mM IPTG and10% n-dodecane or 10% DINP, Data are the means of 3 repetitions ± SD

CymAa and CymAb catalyze the biosynthesis of (*R*)-(+) perillyl alcohol using (*R*)-(+) limonene as a direct substrate through a hydroxylated reaction at the 7 position. We cloned *cymAa* and *cymAb* from *Pseudomonas putida* into pSC04&pSC05. Terpenoid synthase is expressed to low levels in *E. coli* [35], so *cymAa and cymAb* were codon optimized (Table S1). According to the analysis of chirality of the intermediate limonene produced by SC02, we concluded that the configuration of perillyl alcohol was *R*-type (Fig. 3b). Expression levels in the perillyl alcohol pathway for SC04 were investigated by qRT-PCR. The results indicated that *ERG8, ERG19 ERG12,* and *IDI* significantly increase compared to the control. Moreover, changes in the expression of *mvaE, mvaS, CILS, GPPS, cymAa* and *cymAb* were comparable to the control (Fig. 4a). We used n-dodecane or DINP as the extraction solvent and the organic phase was collected and centrifuged to remove cell contaminants. Samples were then subjected to GC-MS analysis and trace levels of perillyl alcohol were detected. pYJM14 and pSC04&pSC05 were co-transformed into *E. coli* BL21 (DE3), resulting in recombinant strain SC04&SC05. SC04 that was cultured in sealed shake flasks to assess perillyl alcohol production. The fermentation broth of the strain SC04 was extracted by ethyl acetate, TLC, product refined and optical rotation detection. The specific rotation value of the product was [α]_D_^20^ = + 82 ± 1 (C 1.8, CHCl_3_), the standard was [α]_D_^20^ = + 84 (C 1.8, CHCl_3_) [36]. From the above, the fermentation product can be identified as (*R*)-(+)-perillyl alcohol.

The (*R*)-(+)-perillyl alcohol levels for SC04 reached 12.5 mg/L after induction by 0.2 mM IPTG and 10% DINP overlay for 48 h, which was 2.4-fold higher than SC05 (7.2 mg/L for 48 h) (Fig. 4b). In the absence of DINP, SC04 and SC05 produced 3.1 mg/L and 1.5 mg/L of (*R*)-(+)-perillyl alcohol. We included n-dodecane overlays in the cultures, and thus a small amount of perillyl alcohol was detected [32]. The production of (*R*)-(+)-perillyl alcohol was 9.3 mg/L in SC04 and 5.6 mg/L in SC05, mainly due to the rapid extraction of limonene to the organic phase, and as n-dodecane overlay could not be used for perillyl alcohol fermentation. In the SC04, the production of (*R*)-(+)- perillyl alcohol and mevalonate was 12.5 mg/L and 102 mg/L after 48 h of induction (BL21 (DE3)/pET-28a(+) not detected, Fig. S1). So, the production of (*R*)-(+)-perillyl alcohol was through the mevalonate pathway from glucose.

#### Fed-batch culture of engineered E.coli strains

**Fig. 5:**
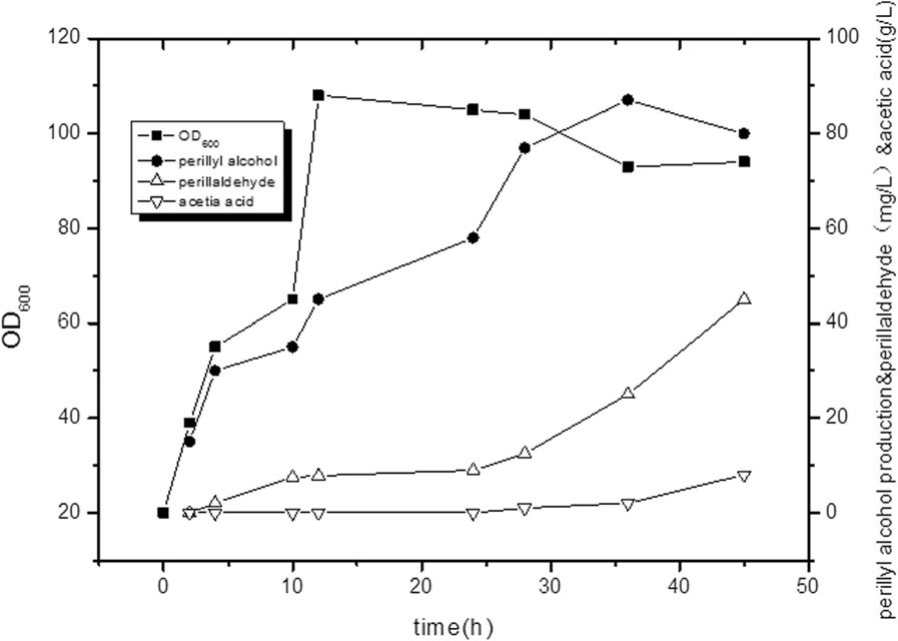
Time-course of (*R*)-(+)-perillyl alcohol production during fed-batch fermentation of SC04

High cell densities for (*R*)-(+)-perillyl alcohol production were achieved through fed-batch fermentation using SC04 in a 5 L bioreactor. According to the residual glucose concentrations, feeding rates were maintained at ≤1 g/L. Figure 5 shows the time-course of (*R*)-(+)-perillyl alcohol production and cell density during fermentation. (*R*)-(+)-perillyl alcohol biosynthesis was initiated from 5 h of induction and 10% DINP was added during the fed-batch process. After 36 h of induction, the production of (*R*)-(+)-perillyl alcohol peaked at 87 mg/L with DINP overlay and the yield was 1.5 mg/g glucose. The theoretical conversion yield of (*R*)-(+)-perillyl alcohol from glucose is 28.1% (1.5 glucose~ 0.5 (*R*)-(+)-perillyl alcohol) However, the engineered strain achieved a cell-density OD_600_ of 108 for two-phase fermentation. In the late fermentation stage, the content of perillyl aldehyde and acetic acid increased to 45 mg/L and 8 g/L respectively, which hindered the production of (*R*)-(+)-perillyl alcohol [37]. The accumulation of IPP and DMAPP may have influenced the growth of the engineered strain [25, 38, 39]. However, the two-phase fermentation of (*R*)-(+)-perillyl alcohol fermentation eliminated toxic intermediates. (*R*)-(+)-perillyl alcohol biosynthesis requires many overexpressed genes, which influence host cell viability and reduce productivity [40].

## Discussion

(*R*)-(+)-perillyl alcohol is produced in perilla leaves, citrus, lemon and lavender and has a range of applications. Many terpenes are synthesized from limonene, such as perillyl alcohol, carvone and α-terpineol. As the chemical synthesis of perillyl alcohol is of high cost and is environmentally unfriendly, the biosynthesis of perillyl alcohol from renewable carbon sources is regarded as an economically feasible industrial process. Indeed, the development of (*R*)-(+)-perillyl alcohol as an anti-cancer drug has been limited by the costs associated with extracting the compound from its natural oil. Terpenes are produced by microorganisms and can be scaled-up to increase drug production [41]. We used *E.coli* as a host and confirmed the feasibility of (*R*)-(+)-perillyl alcohol production. The levels produced were however, too low for industrial requirements.

Through comparison of strain SC00 and SC01, we found that SC00 by expressed *LHBS* from *Bacillus stearothermophilus* BR 388 produced a little, 1.5 mg/L (Fig. 2b), It may be that cofactors (NADPH, ATP) are involved in the fermentation process, which reported the production of 0.51 mg/Lwith (*R*)-(+)-limonene substrate [21]. so we directly selected the *cymAa and cymAb* which convert limonene derived from the metabolism of *p*-cymene [15]. Enzyme activity was lower than other terpene synthases, that reduce perillaldehyde or perillic acid [15]. We initially engineered SC02 and showed successful (*R*)-(+)-perillyl alcohol production using GC-MS (Fig. 2d). The maximum production of (*R*)-(+)-perillyl alcohol composed with (*R*)-(+)-limonene as substrate was 86.9 mg/L and lethal concentrations of limonene to *E. coli* were 2 mM (Fig. 2b). (*R*)-(+)-Limonene accumulates in biological membranes leading to damage. The compound is also insoluble in water [42]. It has been reported that limonene is inhibitory to multiple microorganisms [43]. A recent report revealed that 0.025% (v/v) limonene inhibited the growth of *E. coli* [44]. Thus, the tolerance and solubility of limonene requires improvement to increase both the concentrations and yields of (*R*)-(+)-perillyl alcohol.

In previous studies, terpenoids have been studied using the MVA pathway [23, 25, 26, 45]. Our research group constructed engineered *E.coli* which synthesized isoprene by the mevalonate (MVA) pathway, and produced isoprene to 665.2 mg/L under flask conditions [46, 47]. GPPS and CILS were engineered to improve (*R*)-(+)-limonene production, and SC03 produced no detectable limonene (data not shown), highlighting the need for terpenes. When no extracts were added during limonene fermentation, no detectable SC02 was observed (data not known), indicating that limonene is rapidly lost from the culture medium. As limonene from the fermentation system was highly volatile and anti-microbial [48, 49] other collection methods have been reported including culture extraction, solvent overlay, solid-phase micro-extraction, gas shipping to a cold trap, and adsorbent polydimethlsiloxane bars [48, 50, 51]. Whilst not all of these methods are appropriate, they do prevent product inhibition and toxicity, avoiding evaporative loss of the limonene product using the two-phase system. Davies et al. [52] showed that an overlay of n-dodecane enhanced limonene recovery. N-dodecane was selected as a favorable solvent for terpenoid extraction experiments due to its low volatility [52]. In engineered *E.coli,* DINP fermentation prolongs both the growth and production phases, leading to (*S*)-(−)-limonene concentrations of 1.35 g/L [53]. (*R*)-(+)-limonene obtained by n-dodecane extraction was 45 mg/L for 48 h (Fig. 3c). It may be that the distribution coefficient of (*R*)-(+)-limonene in n-octane is larger than that of the DINP phase. The strain fermented by the DINP overlay also inhibited cell growth, and the OD_600_ decreased from 3.4 to 2.5 (Fig. 3c). Jongedijk et al. [48] engineered *Saccharomyces cerevisiae* to express (*R*)-(+)-limonene synthase from *Citrus limon*. Trapping of the headspace in limonene synthase expressing strains resulted in 0.12 mg/L (*R*)-(+)-limonene. Pang et al. [54] engineered *Yarrowia lipolytica* to achieve (*R*)-(+)-limonene at 11.705 mg/L, through the overexpression of *HMGR* and the optimization of the fermentation conditions. Cao et al. [55] engineered *Yarrowia lipolytica* to increase limonene titers to 23.56 mg/L by encoding neryl diphosphate synthase1 (NDPS1) and limonene synthase (LS). Here, we engineered *E. coli* to produce (*R*)-(+)-limonene to 45 mg/L.

We confirmed that the chirality of perillyl alcohol was (*R*)-type in SC04, which was performed with (*R*)-(+)-limonene as an intermediate (Fig. 3b). We then engineered SC04 & SC05 and investigated the effects of copy numbers on (*R*)-(+)-perillyl alcohol production. We hypothesized that *cymAa* and *cymAb* genes produced by the lower copies of SC05 could not repress all promoters. In contrast, SC04 had 40 copies encoding the repressor from medium and middle-copy plasmids. The genes contributed to higher levels in SC04 than in SC05 (Fig. 4a). This hypothesis was supported by the production of perillyl alcohol, which was 1.7-fold in SC04 and SC05 (from 7.2 mg/L to 12.5 mg/L) (Fig. 4b). In addition, the levels of *ERG12*, *ERG8*, and *ERG19* in the MVA pathway were higher than those of the control strain, whilst the levels of *GPPS*, *ClLS*, *cymAa* and *cymAb* showed no obvious changes compared to controls (Fig. 4a). Most intermediates can be efficiently converted to IPP, but the production from IPP to perillyl alcohol was weak. This suggests the enhanced production of (*R*)-(+)-perillyl alcohol through regulating metabolic imbalances.

(*R*)-(+)-perillyl alcohol production was achieved by assembling biosynthetic genes encoding a heterologous MVA pathway. The highest-performing strain (SC04) accumulated up to 87 mg/L of (*R*)-(+)-perillyl alcohol under fed-batch fermentation conditions. The synthesis of (*R*)-(+)-perillyl alcohol reported in this study has not been systematically optimized. From the perspective of fermentation process (Fig. 5), the low yield of (*R*)-(+)-perillyl alcohol depends on two aspects. On the one hand, the by-product perillaaldehyde leads to the decrease of (*R*)-(+)-perillyl alcohol content. It may be due to the presence of alcohol dehydrogenase [56] (such as *yig*B) which can convert geraniol to geranial in *E. coli*. Therefore, the key gene *cymAa and cymAb* were overexpressed and optimized. On the other hand, the fermentation control was optimized to reduce the content of acetic acid [57] and promote cell growth.

## Conclusions

We have engineered *E. coli* to produce (*R*)-(+)-perillyl alcohol from glucose through the MVA pathway. In this study, it was firstly reported that (*R*)-(+)-perillyl alcohol was synthesized from glucose in recombinant *Escherichia coli*, which produced 87 mg/L of (*R*)-(+)-perillyl alcohol. It provides new methods for the synthesis of chiral perillyl alcohol. Efforts should now be directed towards the optimization of the MVA pathway.

## Methods

### Strains and culture conditions

Metabolic engineering for natural compound production can be enhanced through gene modifications that increase enzyme activity. All experimental materials used in this study are listed in Table S1*. E.coli* were grown in LB (tryptone 10 g/L, yeast extract 5 g/L and NaCl 10 g/L). During the production of (*R*)-(+)-limonene and (*R*)-(+)-perillyl alcohol, strains were cultivated in shake-flasks in medium containing glucose 10 g/L, MgSO_4_ 1 mM and riboflavin 0.05 mM during fed-batch fermentation in glucose 20 g/L, K_2_HPO_4_ 9.8 g/L, ferric ammonium citrate 0.3 g/L, citric acidmonohydrate 2.1 g/L, MgSO_4_ 1 mM, riboflavin 0.05 mM and 1 mL trace element solution, including (NH_4_)_6_Mo_7_O_24·_4H_2_O 0.37 g/L, ZnSO_4·_7H_2_O 0.29 g/L, H_3_BO_4_ 2.47 g/L, CuSO_4·_5H_2_O 0.25 g/L, and MnCl_2·_4H_2_O 1.58 g/L. As required, ampicillin (100 μg/ml) and kanamycin (50 μg/ml) were added for selection.

### Plasmid construction

Limonene hydroxylase (pOT435) gene (*LHBS*, GenBank Accession No. AF039527.1) from *Bacillus stearothermophilus* BR 388, were codon optimized and cloned into pUC57. *LHBS* were PCR amplified and cloned into pET28a (+) with *Eco*I*/Xho*I restriction sites, creating pSC00 (pET28a-LHBS). P-cymene monoxygenase hydroxylase (*cymAa*, GenBank Accession No. AAB62299.1) and P-cymene monoxygenase reductase (*cymAb*, GenBank Accession No.:AAB62300.1) from *Pseudomonas putida* were codon optimized by BGI, and cloned into pUC57. *cymAa* and *cymAb* were PCR amplified and subcloned into pET28a (+) with *BamH*I/*Sac*I restriction sites, creating pSC01 (pET28a-cymAa-cymAb). *ClLS* (GenBank Accession No.:AF514287.1) of *Citrus limon* and *GPPS* (GenBank Accession No.:AF513112.1) of *Abies grandis* were optimized by BGI and synthesized by GeneWiz (Suzhou, China), producing pUC57-ClLS&pUC57-GPPS. MvaE-mvaS was then excised from pYJM20 [46] and ligated into pET-28a (+) to create pET28a-mvaE-mvaS. *GPPS* and *ClLS* which were truncated in the N-terminus, were cloned and assembled into pET28a-mvaE-mvaS at the *Sac*I/*Aat*II sites to generate pSC02 (Table S1). *CymAa-cymAb* fragments were obtained through *Pseudomonas putida* using *Aat*II and *Pac*I and ligated into pSC02 to create pSC03 (Table S1). MvaE, mvaS, and GPPS were cloned from pSC02 into pCOLADuet-1 at the *BamH*I/*Xho*I sites, generating pcolaDuet-mvaE-mvaS-GPPS-ClLS. *CymAa, cymAb* were cloned from pSC03 into pcolaDuet-mvaE-mvaS-GPPS-ClLS at the *Xho*I/*Pac*I sites to produce pSC05 (Table S1).

pYJM14 was constructed from pTrcHis2B through the introduction of *ERG8, ERG12, ERG19* and *IDI* from *S. cerevisiae* [33, 58]. All plasmids and primers are shown in Table S2.

#### RT-PCR analysis

Total RNA was isolated from 24 h cultures using commercially available SPAKeasy RNA kits. RNA was reverse transcribed using TaKaRa Primer Script RT reagent Kit and RT-PCRs were performed. Each reaction contained 1 μL cDNA, 5 μL TB green Premix Ex TaqII, 0.2 μL 50 × ROX Reference Dye, 0.2 μM for/rev primer, and ddH_2_O up to 10 μl. RT-PCR conditions were as follows: 30 s at 95 °C, 40 cycles of 95 °C for 5 s, 60 °C for 30 s. Gene expression was normalized to the absolute transcript levels of *rpoD.* qRT-PCRs were performed on a Primer 5.0 program (Table S3). Relative gene expression was calculated using the 2^ΔΔ^Ct method for each treatment. Reactions were repeated a minimum of 3 times.

#### Shake-flask fermentation

Cultures were produced in in 25 ml of LB. *E. coli* strains with each recombinant plasmid inoculated in a gyratory shaker at 37 °C and 180 rpm. IPTG (0.2 mM) was added to induce recombinant protein expression upon an OD_600_ of 0.6. Cultures were incubated at 30 °C with 2-phase fermentation [59] used for limonene and perillyl alcohol extraction from the aqueous broth due to the toxicity of terpenes. We added 10% (v/v) n-dodecane or DINP (disononyl phthalate) following IPTG induction and cultures were incubated for 48 h. Cell densities, glucose levels, limonene levels and perillyl alcohol production were then assessed.

#### Fed-batch fermentation

For (*R*)-(+)-perillyl alcohol production on a larger scale, fed-batch cultivations were performed in a 5 L bioreactor system (Biostat B plus MO 5 L) using 2 L of fermentation fluid. Seed cultures (100 ml, 10 g NaCl, 5 g yeast extract, and 10 g of tryptone per 1 L) were added to shake flasks overnight at 37 °C and Sparger aeration was performed to maintain high dissolved oxygen (DO) levels. Post-fermentation, the pH of the broth was maintained at 7.0 through ammonia addition. Fermentation was performed during the growth stages under the following conditions: 37 °C, agitation 400 rpm and airflow at 1 L/min. Antifoam 204 was added as required. DO was maintained at 20% saturation through the control of air flow and stirrer speed (1–2 L/min and 400–900 rpm, respectively). When cells reached an OD_600_ of ~ 20, the temperature was switched to 30 °C and 0.2 mM IPTG and 0.05 mM riboflavin were added. DINP (10%) was added after 4 h when the initial glucose levels were exhausted, as indicated by the increase in DO. The fed batch mode was initiated through the feeding of 60% glucose at appropriate rates. Residual glucose levels were maintained to low levels through the addition of acetic acid. Samples were periodically collected and OD_600_ values were determined prior to centrifugation for the separation of the organic and aqueous phases. Organic layers were removed for all GC–MS analysis.

#### Analytical methods

*E. coli* growth was determined through OD_600_ measurements on a spectrophotometer (Cary 50 UV-vis, Varian). The Shimadzu GC-MS system (TQ8050) was used for Limonene and perillyl alcohol identification. GC-MS conditions were as follows: 30 m DB-5MS column (internal diameter 0.32 mm, film thickness 0.25 μm); temperature: 50 °C hold, ramped up 10 °C/min to 250 °C with a final hold at 250 °C for 10 min. Highly pure helium was used as a carrier at a linear velocity of 1 ml/min; an injector temperature of 250 °C; a split ratio of 1:10; an ion source temperature of 230 °C and mass range of *m/z* 40–500. Limonene and perillyl alcohol and perillaldehyde peaks were identified through the retention times of external standards and MS comparisons via the National Institute of Standards and Technology (NIST) database. Fermentation broths were mixed, centrifuged, and the organic layer was taken for GC-MS analysis.

The enantiomeric distribution of limonene was analyzed using the Agilent Technologies 7890B GC System on a Cyclodex B column (30 m × 0.25 mm internal diameter; film thickness = 0.25 μm). GC conditions were as follows: 50 °C hold, 2 °C/min ramping to 160 °C; carrier: high-purity helium, linear velocity: 1 ml/min; temperature of the injector: 250 °C; split ratio: 1:20. Compounds of interest: (*S*)-(−)-limonene at 21.2 min and (*R*)-(+)-limonene at 22.02 min. The fermentation broth was mixed and centrifuged, and the organic layer was analyzed.

The concentration of mevalonate was determined by gas chromatography (GC) analysis on a Aglient19091J-413 HP-5 column (30 m × 0.32 mm internal diameter, film thickness = 0.25 μm). The broth was acidified to pH 2.0 with 3 M HCl and mixed with anhydrous Na_2_SO_4_. The mixture was extracted twice with ethylacetate. Mevalonate in the solvent layers was then analyzed by GC. GC conditions were as follows: 50 °C hold, 10 °C/min ramping to 250 °C, hold 5 min. Carrier: high-purity helium, linear velocity: 1 ml/min; temperature of the injector: 260 °C.

Specific rotation of (*R*)-(+)-perillyl alcohol. The fermentation products were subjected to preparative TLC (PTLC) to isolate pure product [60]. Specific rotation was detected in the automatic polarimeter (JIAHANG). Test conditions: wavelength of sodium light 589 nm, room temperature, length 10 cm, 1.8% (W/V) of CHCl_3_. Three times for each detection.

## Supplementary Information

**Additional file 1: Table S1.** Strains and plasmids used in this study. **Table S2.** Primers used in this study for plasmids construction. The restriction sites in the primers were underlined. **Table S3.** Primers for RT-PCR used in this study. **Fig. S1.** Mevalonate and (*R*)-(+)-perillyl alcohol production engineered MVA pathway. The total production of MVA and (*R*)-(+)-perillyl alcohol was measured after 48 h of IPTG induction.

## Data Availability

The data supporting the conclusions of this article are included within the article.
