# Supplementary material for: Effectiveness of recombinant Escherichia coli on the production of (R)-(+)-perillyl alcohol
Source: BMC Biotechnol. 2021 Jan 8;21:3. doi: 10.1186/s12896-020-00662-7 (PMC7791655; doi:10.1186/s12896-020-00662-7)
Supplement: Supplementary file 1 — Additional file 1: Table S1. Strains and plasmids used in this study. Table S2. Primers used in this study for plasmids construction. The restriction sites in the primers were underlined. Table S3. Primers for RT-PCR used in this study. Fig. S1. Mevalonate and (R)-(+)-perillyl alcohol production engineered MVA pathway. The total production of MVA and (R)-(+)-perillyl alcohol was measured after 48 h of IPTG induction. [file 12896_2020_662_MOESM1_ESM.docx]

***Supplementary material***

# Effectiveness of recombinant *Escherichia coli* for the production of (*R*)-(+)-perillyl alcohol

Chao Sun^1,2^, Xianjuan Dong^2^, Rubing Zhang^2,*^, Congxia Xie^1,*^

^1^A State Key Laboratory Base of Eco-Chemical Engineering, College of Chemistry and Molecular Engineering, Qingdao University of Science and Technology, Qingdao, China

^2^CAS Key Laboratory of Bio-based Materials, Qingdao Institute of Bioenergy and Bioprocess Technology, Chinese Academy of Sciences, Qingdao, China

*Corresponding author.

Rubing Zhang

CAS Key Laboratory of Bio-based Materials, Qingdao Institute of Bioenergy and Bioprocess Technology, Chinese Academy of Sciences,189 Songling Rd., Qingdao 266101, China. E-mail: zhangrb@qibebt.ac.cn

Congxia Xie

A State Key Laboratory Base of Eco-Chemical Engineering, College of Chemistry and Molecular Engineering, Qingdao University of Science and Technology, 53 Zhengzhou Rd., Qingdao 266042, China.

E-mail: xiecongxia@126.com

## Table S1 Strains and plasmids used in this study

| Name | Relevant characteristics | | References |
| --- | --- | --- | --- |
| Strains | | | |
| *E.coli* DH5α | | F^-^ *rec*A *endA*1*Φ80dlac*Z*△M15hs*dR17(r_k_^-^m_k_^+^)λ^-^ | Invitrogen |
| *E.coli* BL21(DE3) | | F- ompT hsdSB (rB – mB - ) gal dcm rne131 λ(DE3) | Invitrogen |
| SC00 | | *E.coli* BL21(DE3) /pSC00 | This study |
| SC01 | | *E.coli* BL21(DE3) /pSC01 | This study |
| SC02 | | *E.coli* BL21(DE3) /pSC02, pYJM14 | This study |
| SC03 | | *E.coli* BL2 (DE3) /pSC03 | This study |
| SC04 | | *E.coli* BL21(DE3) /pSC04, pYJM14 | This study |
| SC05 | | *E.coli* BL21(DE3) /pSC05, pYJM14 | This study |
| Plasmids | | | |
| pTrcHis2B | pBR322 origin, Amp | | Invitrogen |
| pET28a(+) | Kan^r^oripBR322lacI^q^T7p | | Novagen |
| pCOLADuet-1 | Kan^r^ ColA lacI T7lac | | Novagen |
| pYJM14 | pTrcHis2B carrying ERG12, ERG8, ERG19 and IDI from *Saccharomyces cerevisiae* | | [1] |
| pSC00 | pET28a(+) carrying *LHBS* from *Bacillus stearothermophilus* BR388 | | This study |
| pSC01 | pET28a(+) carrying *cymAa* and *cymAb* from *Pseudomonas putida* | | This study |
| pSC02 | pET28a(+) carrying *mvaE* and *mvaS* from *Enterococcus faecalis*, *GPPS* from *Abies grandis*, *ClLS* from *Citrus limon.* | | This study |
| pSC03 | pET28a(+) carrying *GPPS* from *Abies grandis*, *ClLS* from *Citrus limon.* | | This study |
| pSC04 | pET28a(+) carrying *mvaE* and *mvaS* from *Enterococcus faecalis*, *GPPS* from *Abies grandis*, *ClLS* from *Citrus limon,* *cymAa* and *cymAb* from *Pseudomonas putida* | | This study |
| pSC05 | pCOLADuet-1 carrying *mvaE* and *mvaS* from *Enterococcus faecalis*, *GPPS* from *Abies grandis*, *ClLS* from *Citrus limon,* *cymAa* and *cymAb* from *Pseudomonas putida* | | This study |

## Table S2 Primers used in this study for plasmids construction. The restriction sites in the primers were underlined

| Primers | Sequences(5’-3’) |
| --- | --- |
| mvaE-F-BamHI | GGGTCGCGGATCCAGGAGGTAAAAAA |
| mvaE-R | CCTTATATCTCCTTTTATTGTTTTC |
| mvaS-F | AAAGGAGATATAAGGAGGTAAAA |
| mvaS-R-ECORI | AGAGCTCGAATTCTTAGTTTCGATAAGAG |
| GPPS-F-SacI | ACTAAGAATTCGAGCTCTAATACGA |
| GPPS-R | TTATATCTCCTTTTAGTTCTGAC |
| ClLS-F | AGCTACTCATTATATCTCCTTTTAGTTCTG |
| ClLS-R-AaTII | AATTCCCCTATAGTGAGTCGTATTAGACGTCTTAACCTTTGG |
| LHBS-F-EcoRI | CGCGGATCCGAATTCATGGGCAGCAAATAT |
| LHBS-R-XhoI | GGTGGTGGTGCTCGAGTTAACTAA |
| cymAa-F-AaTII | GTTAAGACGTCTAATACGACTCACTATAGGGGAATTGTGAG |
| cymAa-R | GGTCCGAGCCTGGCACGTGGTTAAAAGGAG |
| cymAb-F | GTGGTTAAAAGGAGATATAATGCGCAGCTTTTTTCA |
| cymAb-R-PacI | TGGTGGTGGTGTTAATTAATTAGCTATTACGGCCATCGG |
| mvaE-mvaS-2-F-BamHI | CAGCCAGGATCCGAGGAGGTAAAAA |
| mvaE-mvaS-2-R-SacI | AGGCGCGCCGAGCTCTTAGTTTCGA |
| GPPS-ClLS-2-F-BagII | CATATGGCAGATCTATGGAATTCGACTTCAACAAATAC |
| GPPS-ClLS-2-R-XhoI | ATTCCTATAGTGAGTCGTATTACTCGAGTTAACCTTTGGTGCCCGGACTT |
| cymAa-cymAb-2-F-XhoI | CACCAAAGGTTAACTCGAGTAATACGACTCACTATAGGAATTGTGAGCG |
| cymAa-cymAb-2-R-PacI | CGGTGGCAGCAGCCTAGGTTAATTAATTAGCTATTACGGCCATCGG |

## Table S3 Primers for RT-PCR used in this study

| Primers | Sequences(5’-3’) |
| --- | --- |
| rpoD-F | AGCGTGGAATCTGAAATCGG |
| rpoD-R | GCAACGGAGCATTGAACCTG |
| mvaE-1 | GAGAAAGATGAAGGGATTCG |
| mvaE-2 | CACGATTGAAGTTGCTGC |
| mvaS-1 | CTGAAGCCAGAAATGTAGACC |
| mvaS-2 | AACGATGTAAGACAACTGCG |
| GPPS-1 | GCAAACCCTGGAATGGAT |
| GPPS-2 | TTCAGCAGTTCGTCAGAGAA |
| ClLS-1 | GAAGCCCGTTGGTTTATTC |
| ClLS-2 | CAATGCCCATACTCCACAG |
| cymAa-1 | GGGAACTGAAACTGGATGC |
| cymAa-2 | GGCAATAATGCTCGGAATG |
| cymAb-1 | CTGAGCAAAGCAGAACTGG |
| cymAb-2 | ATTCGCCACCTGGAACTT |
| ERG8-1 | CAGAGTTGAGAGCCTTCAGTG |
| ERG8-2 | ACGATGTTCGGTAACGCTA |
| ERG12-1 | CTGCTAATAAGCGAGTCATCTG |
| ERG12-2 | GTAGTGGAAGGATTCGGATAGT |
| ERG19-1 | GCAAGATGACCTCAGAACG |
| ERG19-2 | TTTCGGAGACAATGTGGAG |


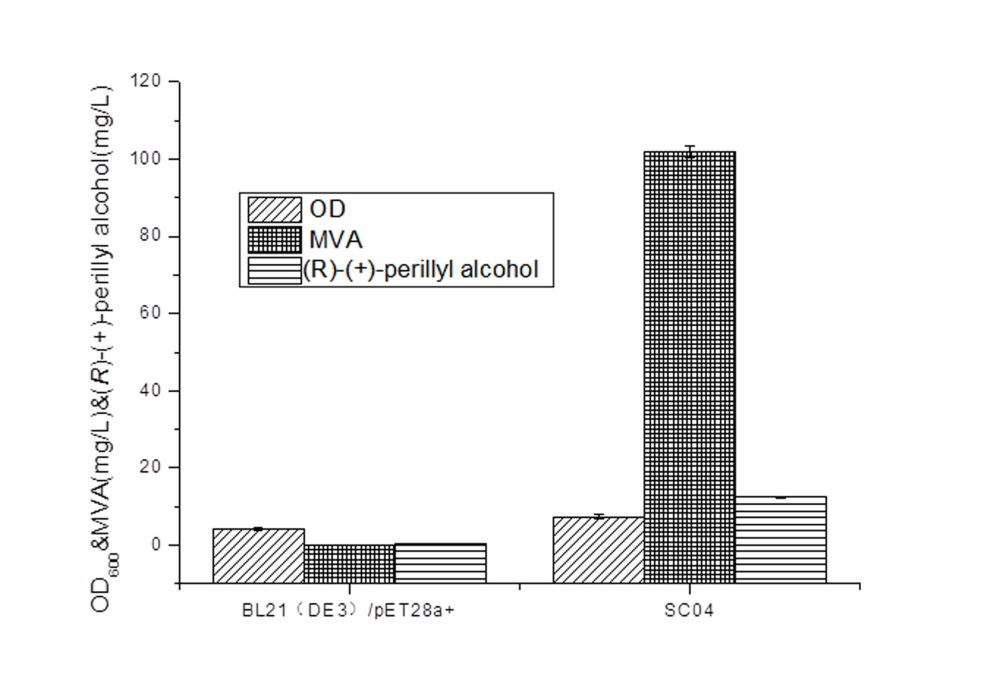


## Figure S1 Mevalonate and (*R*)-(+)-perillyl alcohol production engineered MVA pathway. The total production of MVA and (*R*)-(+)-perillyl alcohol was measured after 48 h of IPTG induction .

1. Yang J, Zhao G, Sun Y, Zheng Y, Jiang X, Liu W, Xian M: Bio-isoprene production using exogenous MVA pathway and isoprene synthase in *Escherichia coli*. Bioresour Technol. 2012, 104:642-647.
